# Matrine attenuates focal cerebral ischemic injury by improving antioxidant activity and inhibiting apoptosis in mice

**DOI:** 10.3892/ijmm.2015.2260

**Published:** 2015-06-24

**Authors:** PENG ZHAO, RU ZHOU, XIAO-YUN ZHU, YIN-JU HAO, NAN LI, JIE WANG, YANG NIU, TAO SUN, YU-XIANG LI, JIAN-QIANG YU

**Affiliations:** 1 Department of Pharmacology, Ningxia Medical University, Yinchuan, Ningxia Hui Autonomous Region 750004, P.R. China; 2 Department of Medical Sci-tech Research Center, Ningxia Medical University, Yinchuan, Ningxia Hui Autonomous Region 750004, P.R. China; 3 Department of Key Laboratory of Hui Ethnic Medicine Modernization, Ministry of Education, Ningxia Medical University, Yinchuan, Ningxia Hui Autonomous Region 750004, P.R. China; 4 Department of Ningxia Key Laboratory of Craniocerebral Diseases of Ningxia Hui Autonomous Region, Ningxia Medical University, Yinchuan, Ningxia Hui Autonomous Region 750004, P.R. China; 5 Department of College of Nursing, Ningxia Medical University, Yinchuan, Ningxia Hui Autonomous Region 750004, P.R. China; 6 Department of Ningxia Hui Medicine Modern Engineering Research Center and Collaborative Innovation Center, Ningxia Medical University, Yinchuan, Ningxia Hui Autonomous Region 750004, P.R. China

**Keywords:** matrine, neuroprotection, cerebral ischemia, apoptosis, oxidative stress

## Abstract

Matrine, an active constituent of the Chinese herb, *Sophora flavescens* Ait., and it is known for its antioxidant, anti-inflammatory and antitumor activities. It has been demonstrated that matrine exerts protective effects against heart failure by decreasing the expression of caspase-3 and Bax, and increasing Bcl-2 levels. In this study, we aimed to determine whether these protective effects of matrine can be applied to cerebral ischemia. Following 7 successive days of treatment with matrine (7.5, 15 and 30 mg/kg) and nimodipine (1 mg/kg) by intraperitoneal injection, male Institute of Cancer Research (ICR) mice were subjected to middle cerebral artery occlusion (MCAO). Following reperfusion, the neurobehavioral score and brain infarct volume were estimated, and morphological changes were analyzed by hematoxylin and eosin (H&E) staining and electron microscopy. The percentage of apoptotic neurons was determined by flow cytometry. The levels of oxidative stress were assessed by measuring the levels of malondialdehyde (MDA), superoxide dismutase (SOD), glutathione peroxidase (GSH-Px) and catalase (CAT), and the total antioxidant capacity (T-AOC). Western blot analysis and immunofluorescence staining were used to examine the expression of the apoptosis-related proteins, caspase-3, Bax and Bcl-2. Our results revealed that pre-treatment with matrine significantly decreased the infarct volume and improved the neurological scores. Matrine also reduced the percentage of apoptotic neurons and relieved neuronal morphological damage. Furthermore, matrine markedly decreased the MDA levels, and increased SOD, GSH-Px and CAT activity, and T-AOC. Western blot analysis and immunofluorescence staining revealed a marked decrease in caspase-3 expression and an increase in the Bcl-2/Bax ratio in the group pre-treated with matrine (30 mg/kg) as compared with the vehicle-treated group. The findings of the present study demonstrate that matrine exerts neuroprotective effects against cerebral ischemic injury and that these effects are associated with its antioxidant and anti-apoptotic properties.

## Introduction

Stroke remains a leading cause of death and long-term disability worldwide, thus representing a major concern to public health and the economy (1). According to statistics, 60–70% of all stroke victims suffer an ischemic stroke (2,3), which is characterized by the occlusion of blood vessels by the formation of an obstructive thrombus or embolus in the brain, consequently resulting in an inadequate supply of blood and oxygen to the brain (4). Re-establishment of blood circulation to the ischemic brain as soon as possible is the most effective therapy for patients with ischemic stroke. However, reperfusion itself has a latent risk, as it can cause further damage to brain tissue, such as hemorrhagic transformation, cerebral edema, blood-brain barrier (BBB) leakage and neuronal death (3,5). This phenomenon is termed cerebral ischemia/reperfusion (I/R) injury, which is accompanied by a cascade of mechanisms, including glutamate excitotoxicity, calcium overload, oxidative stress, inflammation and apoptosis, eventually leading to cell death (6,7). However, due to the complex mechanisms involved in the I/R process, to date, the only effective American Food and Drug Administration (FDA)-approved pharmacological treatment for ischemic stroke is the intravenous recombinant tissue plasminogen activator (rtPA), the effectiveness of which is extremely limited in clinical therapeutics, owing to the short therapeutic window and an increased risk of subarachnoid hemorrhage (8). Therefore, the investigation of the pathological mechanisms and the search for and development of safe and effective neuroprotective agents for the treatment of ischemic stroke is of critical clinical significance.

**Figure 1:**
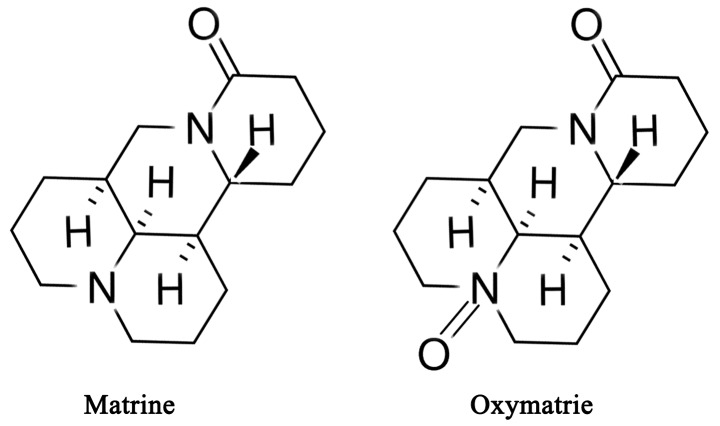
Structure of matrine (Mat) and oxymatrine.

In recent years, natural products, due the abundant resources, multi-targeted mechanisms of activity, few side-effects, and no drug resistance have been increasingly used, such as aloperine (9). Thus, natural products used in traditional Chinese medicine have received increasing attention and their use has been investigated in cerebral I/R injury, revealing the neuroprotective effects of these products (3,10,11). Matrine (Mat; C_15_H_24_N_2_O) and oxymatrine (C_15_H_24_N_2_O_2_), as the main alkaloids extracted from the traditional Chinese herb, *Sophora flavescens* Ait., have been shown to possess a similar molecular structure (Fig. 1) and have been shown to have a variety of pharmacological activities, such as antitumor (12), antioxidant, anti-inflammatory (13) and antiviral properties (14). It has been demonstrated that Mat not only reduces brain edema induced by focal cerebral ischemia (15), but directly protects neurons and astrocytes against focal cerebral ischemia through the inhibition of the nuclear factor (NF)-κB signaling pathway (16). Studies have also indicated that oxymatrine exerts protective effects against myocardial ischemic injury (17), as well as against liver and intestinal I/R injury in animal models (18,19), and that exerts neuroprotective effects against focal cerebral ischemia through the regulation of Bcl-2/Bax expression and the inhibition of caspase-3 activation in ischemic brain tissue (20,21). Furthermore, it has been reported that Mat exerts protective effects against heart failure by inhibiting the upregulation of Bax, caspase-3 and increasing the expression of Bcl-2 in rats (22). However, whether Mat directly protects ischemic neurons against damage by inhibiting the overexpression of caspase-3 and modulating the Bcl-2/Bax ratio in ischemic stroke has not yet been addressed. Thus, the present study was undertaken to assess neuroprotective potential and possible mechanisms of action of Mat by detecting the activities of oxygen radical scavenging enzymes and the expression of the apoptosis-associated proteins, caspase-3, Bax and Bcl-2, in a mouse model of focal cerebral I/R injury induced by middle cerebral artery occlusion (MCAO).

## Materials and methods

### Experiment animals

Male, Institute of Cancer Research (ICR) mice (n=108) weighing between 20.0 to 25.0 g were obtained from the Experimental Animal Center of Ningxia Medical University, Yinchuan, China (certificate no. SYXK Ningxia 20050001). The animals were housed in a temperature-controlled environment (22–24°C) under a 12 h light and dark cycle and had access to food and water *ad libitum*. The experiments were performed as approved by the Institutional Animal Ethics Committee of Ningxia Medical University. This study complied with the internationally accredited guidelines and ethical regulations on animal research. All surgerical procedures were performed under chloral hydrate anesthesia, and all efforts were made to minimize suffering.

### Drug administration

Mat (purity ≥98.0%) and nimodipine were purchased from the Ningxia Institute of Materia Medica, Yinchuan, China and Bayer Pharma AG, Berlin, Germany, respectively. Both compounds were dissolved in saline solution (0.9% NaCl) and injected by an intraperitoneal (i.p.) injection in an application volume of 0.1 ml/10 g body weight and administered 15 min prior to testing for 7 consecutive days.

The mice were randomly assigned into the following 6 experimental groups (n=42 in each group): i) the sham-operated group (sham); ii) the vehicle-treated group (vehicle); iii) the MCAO + Mat (L) group (low dose, L = 7.5 mg/kg); iv) the MCAO + Mat (M) group (medium dose, M = 15 mg/kg); v) the MCAO + Mat (H) group (high dose, H = 30 mg/kg); and vi) the MCAO + nimodipine group (nimodipine = 1 mg/kg).

Mat and nimodipine were administered by an i.p. injection for 7 consecutive days prior to MCAO. The sham and vehicle groups were treated with physiological saline under the same conditions.

### Mouse model of MCAO

Focal cerebral ischemia was induced using the intraluminal filament technique as previously described by Longa *et al* (23). The mice in the sham-operated group were not subjectd to MCAO. Briefly, male mice were anesthetized with an intraperitoneal injection of 3.5% chloral hydrate. Via a midline neck incision, the left common carotid artery (CCA), external carotid artery (ECA) and the internal carotid artery (ICA) were surgically exposed. The ECA was then isolated and ligated. A 4-0 monofilament nylon suture was inserted into the ICA through the ECA to occlude the origin of the left middle cerebral artery (MCA), almost 15–17 mm from the carotid bifurcation. At 2 h following ischemia, the filament was removed for reperfusion. The sham-operated group mice were subjected to the same surgical procedure, but the MCA was not occluded.

### Evaluation of neurological deficits

Neurological deficit scores were evaluated by an examiner who was blinded to the experimental groups at 24 h after MCAO, following a grading system carried out according to a five-point scale (24) as follows: no neurological deficits, 0; unable to extend the contralateral forelimb fully, 1; circling to the contralateral side, 2; falling to the contralateral side, 3; unable to walk spontaneously and depression of consciousness, 4. The higher the neurological deficit score, the more severe the impairment of motor motion injury.

### Measurement of infarct volume

After neurological scoring, 6 rats (randomly selected) from each group were decapitated to remove the brain. The brains were cut into 5 coronal sections (1-mm-thick each) and stained with a 2% solution of 2,3,5-triphenyltetrazolium chloride (TTC) (Sigma, St. Louis, MO, USA) at 37°C for 20 min, followed by fixation with 4% formaldehyde solution overnight. The infarct volumes were calculated using microscope image-analysis software (Image-Pro Plus; Media Cybernetics, Rockville, MD, USA). To compensate for the effects of brain edema, the exact infarct volumes were calculated according to the following equation: percentage of corrected infarct volume = (normal hemisphere volume − non-infarct volume of infarct side)/normal hemispheric volume ×100.

### Histopathological analysis

After 2 h of ischemia followed by 24 h of reperfusion, the mice (n=6 from each group) were anesthetized with 3.5% chloral hydrate, and perfused with physiological saline via an aortic root catheter until the liver appeared to be white, followed by 4% paraformaldehyde solution that had been cooled to 4°C. The brains were removed and post-fixed in 4% formaldehyde solution overnight at 4°C. After being dehydrated and embedded with paraffin, the brain tissues were cut into 5-*µ*m-thick coronal sections. The paraffin sections were deparaffined in xylene and rehydrated in gradient ethanol from 100 to 70%. Finally, they were stained with hematoxylin and eosin (H&E). The histopathological changes of the cortex were observed under a light microscope (Olympus, Tokyo, Japan) at a magnification of ×400 and images were acquired.

### Morphological evaluation by electron microscopy

The parietal cortex of the ischemic hemisphere of the mice (n=6 from each group) was collected and fixed for 2 h at 4°C with 2.5% glutaraldehyde, immediately following rinsing with phosphate-buffered saline (PBS) and soaking in 2% osmium tetroxide. This was followed by dehydration and embedding in epon. Ultrathin (60-nm-thick) sections were cut using a diamond knife and placed onto colloid-coated copper grids, and finally, were stained with 0.4% uranyl acetate and 2% lead citrate. The morphological changes of the neurons were then observed and photographed using a transmission electron microscope (H-7650; Hitachi, Tokyo, Japan).

### Determination of indicators of oxidative stress

Following MCAO, the mice (n=6 from each group) were decapitated, and the parietal cortexes of the ischemic hemisphere were quickly removed and washed in chilled saline, and were then homogenized in ice-cold saline (9 volumes) for 20 min to prepare a 10% (w/v) homogenate. The homogenate was centrifuged at 3,500 rpm and 4°C for 15 min. The level of malondialdehyde (MDA), as well as the activities of superoxide dismutase (SOD), glutathione peroxidase (GSH-Px) and catalase (CAT), and the total antioxidant capacity (T-AOC) in the supernatant were investigated using a microplate reader (1510; Thermo Fisher Scientific, Waltham, MA, USA) according to the instructions provided with the assay kits (Nanjing Jiancheng Bioengineering Institute, Nanjing, China). The assay results were normalized to the protein concentration in each sample, and expressed as U/mg protein or nmol/mg protein.

### Apoptosis assay by flow cytometry

Following 24 h of reperfusion, the ischemic penumbra areas of the parietal cortex of the mice (n=6 from each group) was taken removed and place on ice, shredded and treated with trypsin at 37°C for 15 min. This was followed by washing with ice-cold PBS, and filtering through a 400 mesh nylon net 2 times to yield a single cell suspension. The cell suspension was stained with Annexin V-FITC staining solution at 4°C for 15 min, followed by propidium iodide staining solution at 4°C for 5 min in the dark. The samples were then immediately analyzed using a flow cytometer (BD Biosciences, San Jose, CA, USA) and the data were analyzed using CellQuest Pro software.

### Immunofluorescence staining

Paraffin-embedded coronal brain sections (5-*µ*m-thick) were subjected to deparaffinization, rehydration and then to microwave irradiation antigen retrieval (microwave method). The sections (n=6 from each group) were then incubated with the appropriate primary antibodies: caspase-3 (19677-1-AP), Bax (50599-2-lg) and Bcl-2 (12789-1-AP) (caspase-3, 1:50; Bax, 1:50; Bcl-2, 1:50; Proteintech Group, Chicago, IL, USA) overnight at 4°C. The following day, the brain sections were rinsed with cold PBS in order to remove the unbound antibodies and incubated with FITC-labeled goat anti-rabbit IgG (SA00003-2; 1:200; Proteintech Group) for 1 h at room temperature followed by 4′,6-diamidino-2-phenylindole (DAPI) for 5 min at room temperature. Finally, the mean density of Bax, Bcl-2 and caspase-3 in the mouse brains (per section; ×400 magnification) was measured using microscope image-analysis software (Image-Pro Plus; Media Cybernetics) by a single investigator who was blind to the sample identity.

### Western blot analysis

After 24 h of reperfusion, the mice (n=6 from each group) were selected randomly and decapitated, adn the ischemic penumbra areas of the parietal cortex were rapidly collected onto ice and kept at −20°C. The prepared brain tissues were weighed and homogenized in 1:10 (w/v) ice-cold whole cell lysis buffer (Nanjing KeyGen Biotech Co., Ltd., Nanjing, China) using a glass homogenizer. Soluble proteins were collected and centrifuged at 12,000 × g for 10 min at 4°C. Tissue total protein concentrations were determined by a BCA Protein assay reagent kit (Beijing TransGen Biotech Co., Ltd.). Tissue total protein (50 *µ*l; caspase-3, Bax and Bcl-2) was separated by 12% sodium dodecyl sulfate-polyacrylamide gel electrophoresis (SDS-PAGE) and then transferred onto a nitrocellulose membrane. The membrane was blocked with PBST containing 5% non-fat dry milk for 1 h, and then incubated overnight at 4°C with the corresponding primary antibodies (Bax, 1:500; Bcl-2, 1:500; caspase-3, 1:1,000; Proteintech Group). After washing 3 times with PBST, the membrane was incubated with secondary antibody (anti-rabbit IgG, SA00001-2; 1:3,000; Proteintech Group). An anti-actin antibody (20536-1-AP; 1:1,000; Proteintech Group) served as the control. The protein band was visualized using an enhanced chemiluminescence (ECL) kit and the density of each band was quantified using a western blotting detection system (Quantity One software; Bio-Rad Laboratories, Hercules, CA, USA).

### Statistical analysis

All statistical analyses were performed using SPSS 17.0 statistical software (SPSS Inc., Chicago, IL, USA). The results are expressed as the means ± standard deviation. The statistical significance of the differences between the various groups was assessed using one-way analysis of variance (ANOVA) followed by the LSD post hoc test. Data of 2 groups were analyzed by an unpaired t-test. Differences were considered statistically significant at values of p<0.05.

## Results

### Mat exerts neuroprotective effects against cerebral ischemia Infarct volume

**Figure 2:**
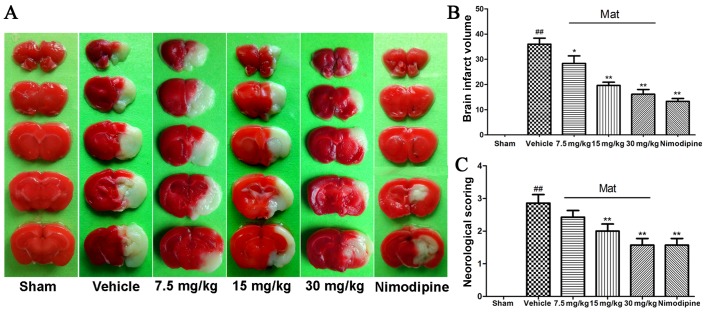
Protective effects of matrine (Mat) against cerebral ischemic injury in mice. (A) 2,3,5-Triphenyltetrazolium chloride (TTC) staining of representative coronal sections at 24 h following reperfusion. (B) Quantitative analysis of the infarct volume at 24 h following reperfusion. (C) Quantification of neurological deficit scores at 24 h following reperfusion. Data are expressed as the means ± SEM (n=6). ^##^p<0.01 vs. sham-operated group; ^*^p<0.05 and ^**^p<0.01 vs. vehicle-treated group.

As shown form the images of the TTC-stained brain sections (Fig. 2A), the infarcted brain tissue appeared white, whereas the normal region appeared red. No infarction was observed in the sham-operated group and an extensive infract area (36.01±5.33%) was observed in the vehicle-treated group. The administration of Mat (7.5, 15 and 30 mg/kg) and nimodipine significantly decreased the percentage of the infarct area to 28.39±6.65% (p<0.05), 19.62±2.85% (p<0.01), 15.76±3.60 % (p<0.01) and 13.31±2.58% (p<0.01), respectively (Fig. 2B).

### Neurological deficit scores

The examination of neurological function was carried out on the mice subjected to 2 h of ischemia followed by 24 h of reperfusion. Compared with the sham-operated group, the neurological deficits were significantly increased in the vehicle-treated group (p<0.01). However, the neurological deficit scores were markedly reduced in the groups treated with Mat (7.5, 15 and 30 mg/kg) and nimodipine (p<0.01). The range in the neurological deficit scores for the different groups is shown in Fig. 2C.

### Histopathological examination

**Figure 3:**
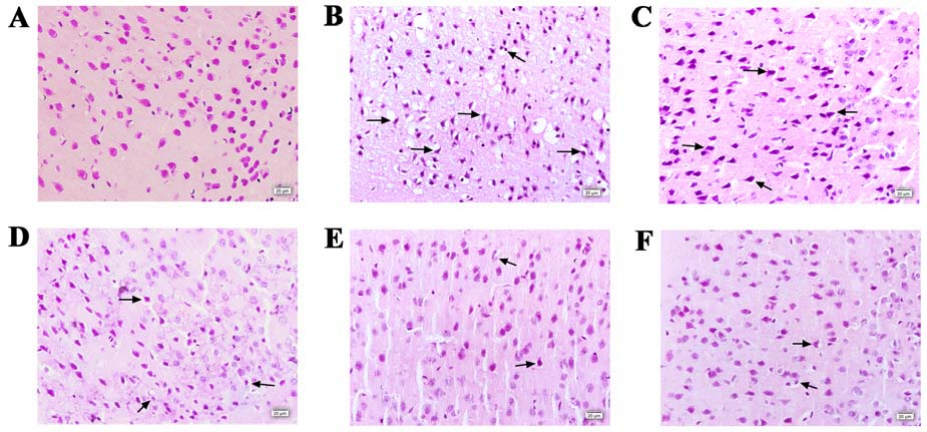
Effects of matrine (Mat) pre-treatment on histological alterations in the ischemic cerebral cortex at 24 h following reperfusion (hematoxylin and eosin staining, ×400 magnification). (A) Sham-operated group. (B) Vehicle-treated group. (C) Mat 7.5 mg/kg-treated group. (D) Mat 15 mg/kg-treated group. (E) Mat 30 mg/kg-treated group. (F) Nimodipine 1 mg/kg-treated group. Arrows indicate necrotic changes with neurons having a scalloped shrunken form in the ischemic lesions.

H&E staining was performed to observe the histopathological changes in the neurons of the mouse brains in the different groups (Fig. 3). In the sham-operated group, the cortex tissue remained intact and the neurons remained well-arranged, and the nuclei were centered with clear staining. However, in the vehicle-treated group (Fig. 3B), a large number of neurons appeared shrunken, swollen, and karyopyknosis and interstitial edema were observed. In addition, neuron arrangement was disordered with loosened and vacuolar neural fibers. However, in the groups pre-treated with Mat and nimodipine, the extent of damage was significantly alleviated, and the number of normal neurons was also markedly increased.

### Morphological evaluation

**Figure 4:**
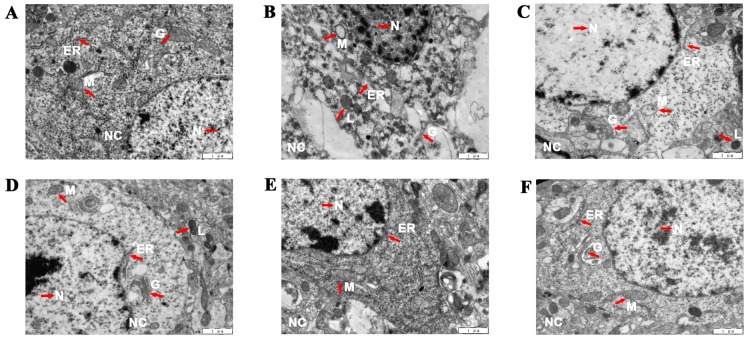
Ultrastructural changes induced by cerebral ischemia and inhibition by matrine (Mat) (×3,000 magnification). (A) Sham-operated group. In the hippocampus, the nerve cell (NC) shows a normal ultrastructure. The nucleus (N), granular endoplasmic reticulum (ER), mitochondrion (M), and Golgi apparatus (G) are indicated. (B) Vehicle-treated group. In the hippocampus, the nerve cell exhibits nuclear chromatine clumping, enlargement of granular ER cisternae, increase in lysosomes (L) and cytoplasmic blebbing. The Golgi apparatus (G) and mitochondrion (M) are indicated. (C–F) Mat (7.5, 15 and 30 mg/kg)- and nimodipine-treated group, respectively. In the hippocampus, the nerve cell (NC) shows nuclear (N) chromatine clumping and slight dilatation of the granular ER cisternae and mitochondrion (M). Lysosomes (L) are indicated. In the Mat 30 mg/kg- and nimodipine-treated groups, the nerve cells (NC) had a relatively normal ultrastructure.

We evaluated the ultrastructural changes of the cortex neurons by transmission electron microscopy, and images were acquired (Fig. 4). The normal cortex neuron contained a large round or oval nucleus with a clear and integrated double nuclear membrane with homogeneous euchromatin and abundant cellular organelles. After 24 h of reperfusion, the cortex neurons showed severe damage. The majority of the nuclei were irregular rather than round or oval in shape with uneven chromatin and a damaged double nuclear membrane, and swollen or vacuolated cellular organelles were observed in the vehicle-treated group (Fig. 4B). By comparison, the morphology of the cortex neurons in the groups pre-treated with Mat and nimodipine showed varying degrees of recovery, displaying relatively normal nuclear membranes, a regular-shaped nucleus and slightly broken cellular organelles.

All of these results demonstrated that Mat alleviated cerebral I/R injury. Moreover, these observations indicated that the most prominent protective effects were observed in the MCAO + Mat (H) (H = 30 mg/kg) group.

### Antioxidant activity of Mat in mice with cerebral I/R injury

**Figure 5:**
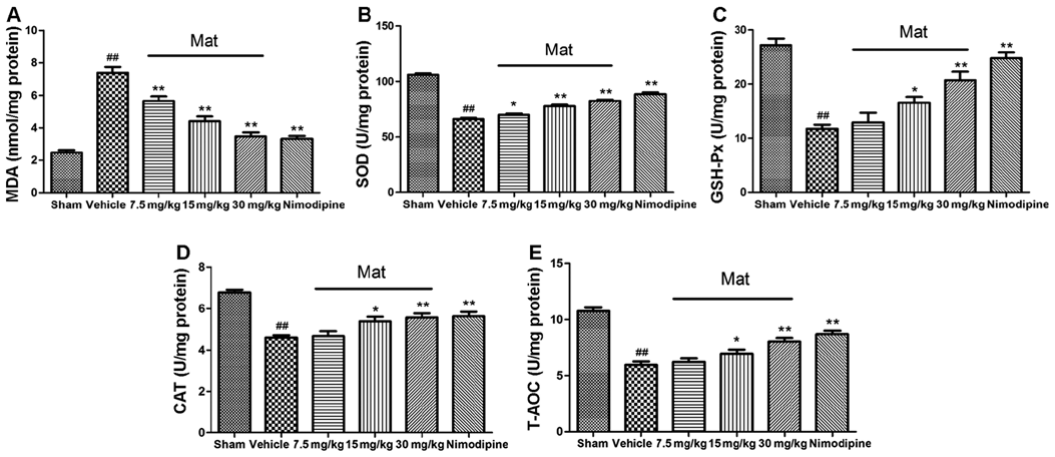
Matrine (Mat) attenuates oxidative stress following focal cerebral ischemia. (A) Effect of Mat on the content of malondialdehyde (MDA) at 24 h following reperfusion. (B) Effect of Mat on the activity of superoxide dismutase (SOD) at 24 h following reperfusion. (C) Effect of Mat on the glutathione peroxidase (GSH-Px) level at 24 h following reperfusion. (D) Effect of Mat on the catalase (CAT) level at 24 h following reperfusion. (E) Effect of Mat on the total antioxidant capacity (T-AOC) at 24 h following reperfusion. Data are expressed as the means ± SEM (n=6). ^##^p<0.01 vs. sham-operated group; ^*^p<0.05 and ^**^p<0.01 vs. vehicle-treated group.

To evaluate the effects of Mat on oxidative stress induced by MCAO, the levels of MDA, as well as the activity of SOD, GSH-Px and CAT, and T-AOC were measured after 24 h of reperfusion. When compared with the sham-operated group, SOD, GSH-Px and CAT activity, and T-AOC were markedly reduced (p<0.01) and the MDA concentration increased significantly (p<0.01) in the vehicle-treated group. Pre-treatment of the mice with Mat exerted antioxidant effects as evidenced by the resoration of SOD, GSH-Px and CAT activity, and T-AOC (Fig. 5B–E) and a decrease in the MDA levels (p<0.05 and p<0.01; Fig. 5A) in a dose-dependent manner after 24 h of reperfusion. Nimodipine produced similar effects.

### Effect of Mat on neuronal apoptosis in mice with cerebral I/R injury

**Figure 6:**
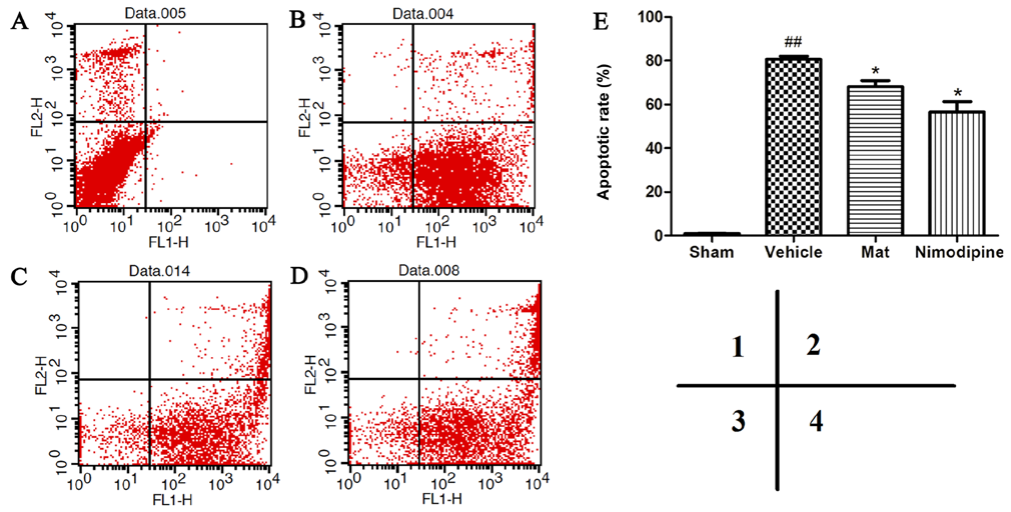
Apoptosis in the ischemic brain cortex neurons was measured by flow cytometry (quadrants 1, 2, 3 and 4 represent dead neurons, late apoptotic neurons, normal neurons and early apoptotic neurons, respectively). (A–D) Apoptotic neurons in the sham-operated group, vehicle-treated group, matrine (Mat) (30 mg/kg)-treated group and nimodipine-treated group, respectively. (E) The percentage of apoptotic neurons in the different groups at 24 h following reperfusion. Data are expressed as the means ± SEM (n=6). ^##^p<0.01 vs. sham-operated group; ^*^p<0.05 vs. vehicle-treated group.

In order to assess cortex neuronal apoptosis, flow cytometry was carried out. After 24 h of reperfusion, the results revealed that, in the sham-operated group, there was a small amount of apoptotic cells in the left cortex. Compared to the sham-operated group, the cortex neuronal apoptotic rate was significantly increased following MCAO (p<0.01). However, the increase in neuronal apoptosis following MCAO was markedly reduced in the groups pre-treated with Mat (H) and nimodipine (p<0.05; Fig. 6).

### Mat affects the expression of apoptosis-associated proteins

**Figure 7:**
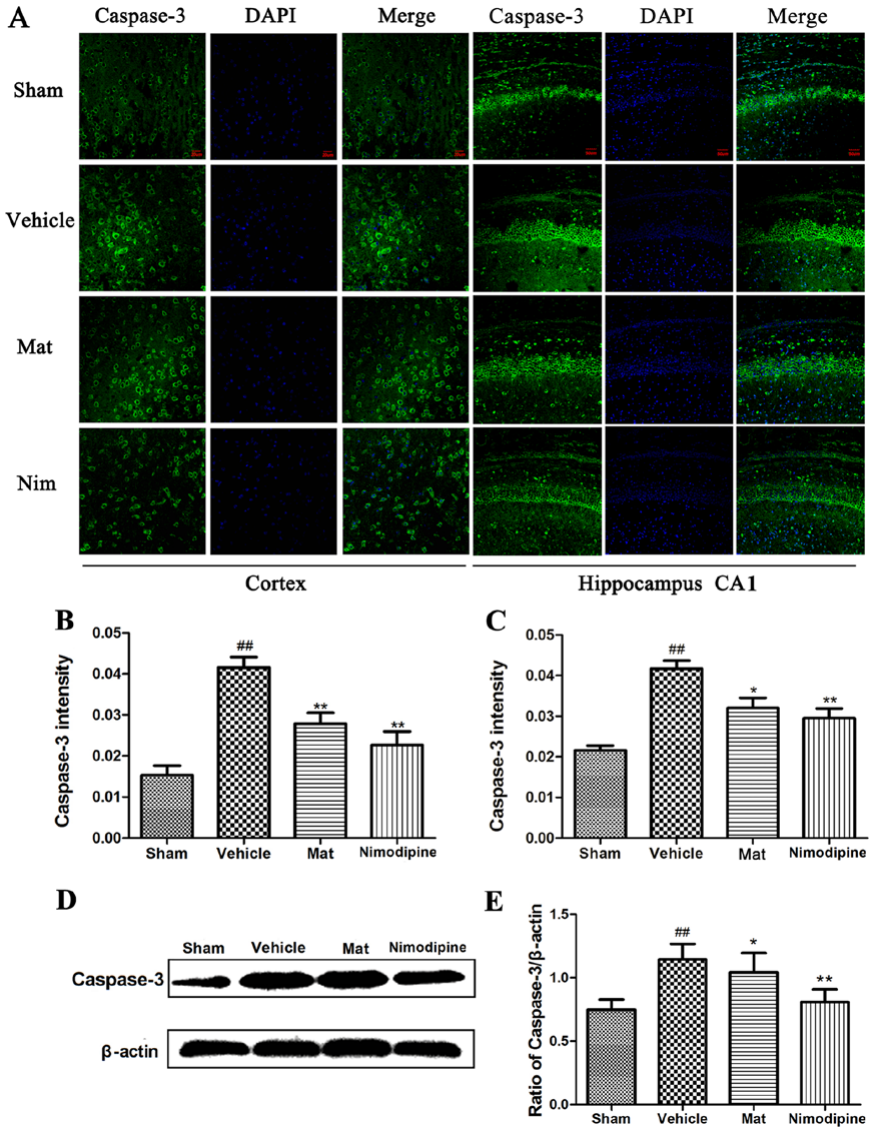
Effects of matrine (Mat) on the expression of caspase-3. (A) Representative photomicrographs of caspase-3 immunofluorescence staining in the ischemic cortex (×400 magnification) and hippocampus CA1 region (×200 magnification). (B and C) Quantification of caspase-3 fluorescence intensity in the ischemic cortex and hippocampus CA1 region in the different groups. (D) Representative western blot of caspase-3 activation in the ischemic cortex at 24 h following reperfusion. (E) Effect of Mat (30 mg/kg) on caspase-3 activation in the cortext in mice subjected to middle cerebral artery occlusion (MCAO) at 24 h following reperfusion. Data are expressed as the means ± SEM (n=6). ^##^p<0.01 vs. sham-operated group; ^*^p<0.05 and ^**^p<0.01 vs. vehicle-treated group.

**Figure 8:**
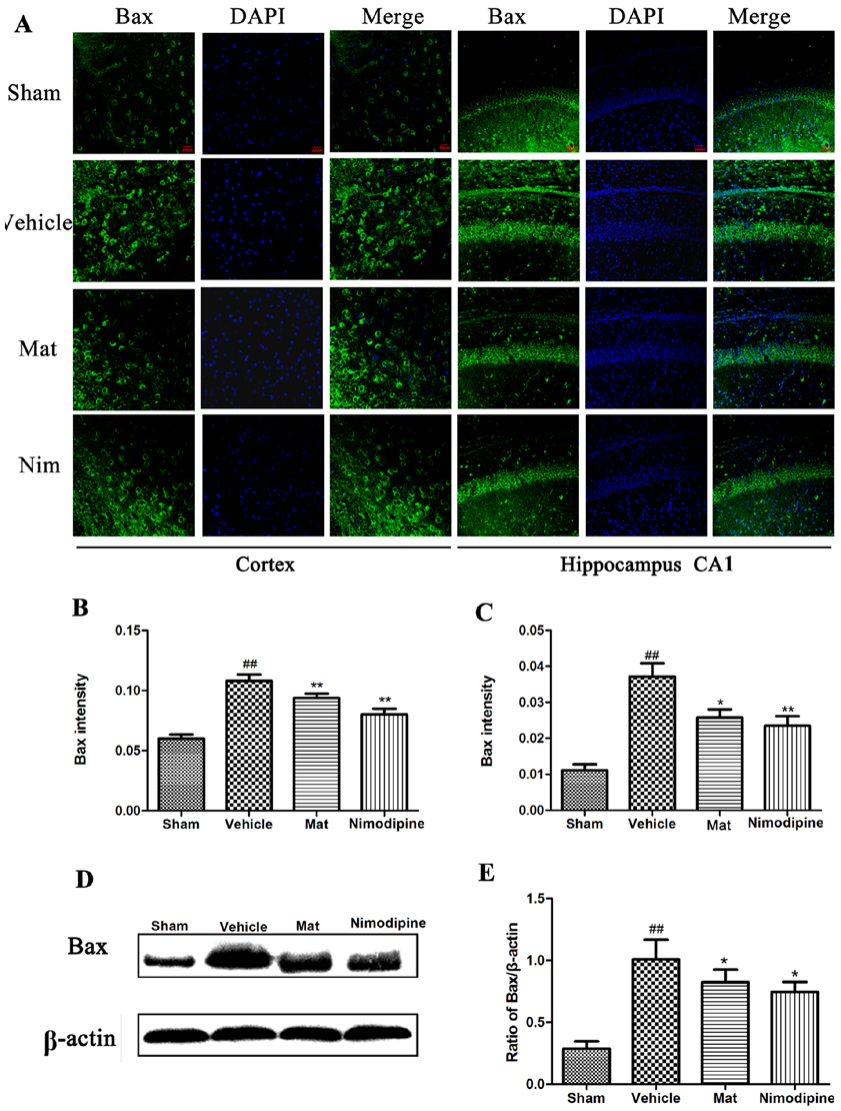
Effects of matrine (Mat) on the expression of Bax. (A) Representative photomicrographs of Bax immunofluorescence staining in the ischemic cortex (×400 magnification) and hippocampus CA1 region (×200 magnification). (B and C) Quantification of Bax fluorescence intensity in the ischemic cortex and hippocampus CA1 region in the different groups. (D) Representative western blot of Bax activation in the ischemic cortex at 24 h following reperfusion. (E) Effect of Mat (30 mg/kg) on Bax activation in the cortext of mice subjected to middle cerebral artery occlusion (MCAO) at 24 h following reperfusion. Data are expressed as the means ± SEM (n=6). ^##^p<0.01 vs. sham-operated group; ^*^p<0.05 and ^**^p<0.01 vs. vehicle-treated group.

**Figure 9:**
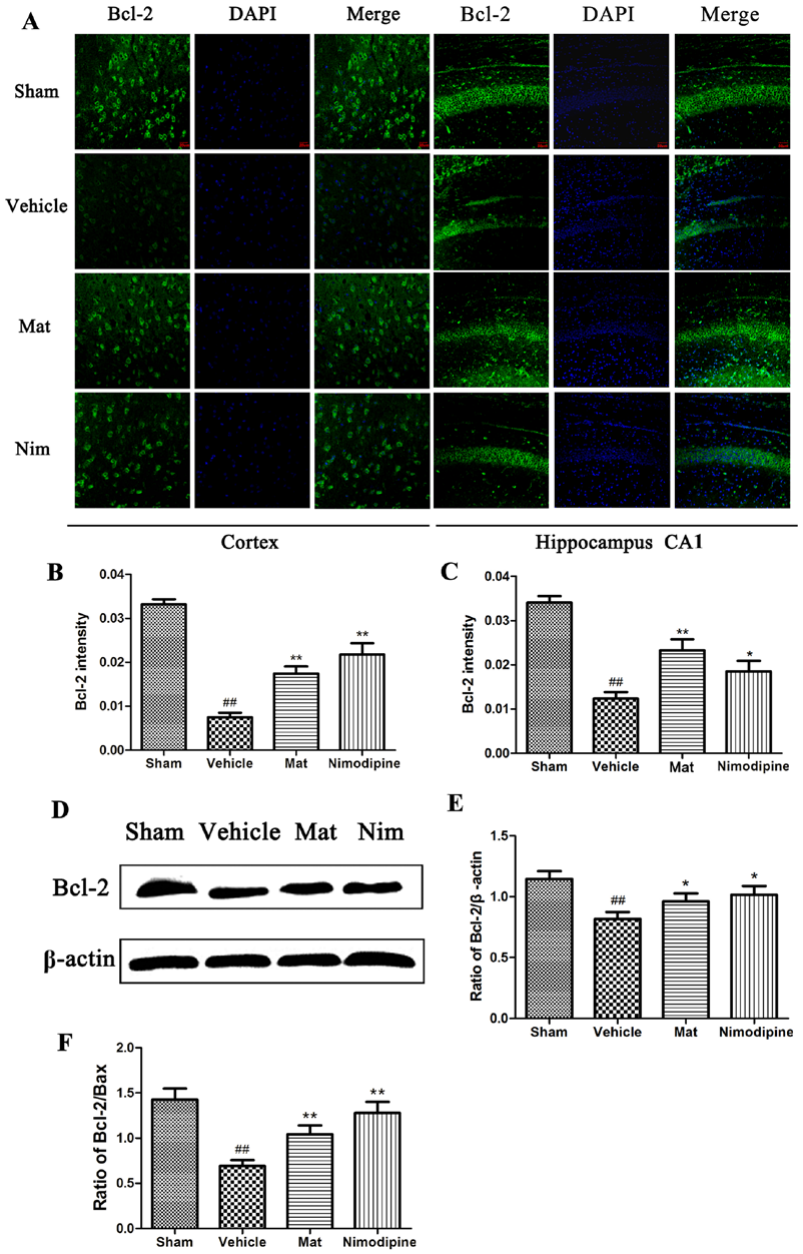
Effects of matrine (Mat) on the expression of Bcl-2. (A) Representative photomicrographs of Bcl-2 immunofluorescence staining in the ischemic cortex (×400 magnification) and hippocampus CA1 region (×200 magnification). (B and C) Quantification of Bcl-2 fluorescence intensity in the ischemic cortex and hippocampus CA1 region in the different groups. (D) Representative western blot of Bcl-2 activation in the ischemic cortex at 24 h following reperfusion. (E) Effect of Mat (30 mg/kg) on Bcl-2 activation in the cortex of mice subjected to middle cerebral artery occlusion (MCAO) at 24 h following reperfusion. (F) Effect of Mat (30 mg/kg) on the Bcl-2/Bax ratio in the cortext of mice subjected to MCAO at 24 h following reperfusion. Data are expressed as the means ± SEM (n=6). ^##^p<0.01 vs. sham-operated group; ^*^p<0.05, and ^**^p<0.01 vs. vehicle-treated group.

To investigate the mechanisms through which Mat inhibits MCAO-induced neuronal apoptosis, the expression of the apoptosis-related proteins, Bax, Bcl-2 and caspase-3, in the hippocampus CA1 and cortex regions of the mice were examined by immunofluorescence staining and western blot analysis. As shown in the images of immunofluorescence staining (Fig. 7A), compared with the sham-operated group, the fluorescence intensity measurements of the protein expression levels of caspase 3 were significantly greater following MCAO (p<0.01). Pre-treatment with Mat (H) and nimodipine significantly reduced the intensity of caspase-3 protein expression (p<0.05 and p<0.01; Fig. 7B and C) in comparison to the vehicle-treated group. On the other hand, compared with the sham-operated group, the vehicle-treated group displayed a higher fluorescence intensity of Bax (Fig. 8A) and a lower fluorescence intensity of Bcl-2 protein (Fig. 9A). However, pre-treatment with Mat (H) or nimodipine resulted in a significant increase in Bcl-2 expression (p<0.05 and p<0.01; Fig. 9B and C) and a marked decrease in Bax expression (p<0.05 and p<0.01; Fig. 8B and C). In line with the results from immunofluorescence staining, the results from western blot analysis demonstrated that the caspase-3 protein level in the vehicle-treated group was markedly increased compared to that of the sham-operated group (p<0.01; Fig. 7D and E). The groups treated with Mat (H) and nimodipine showed a significantly reduced protein expression of caspase-3 in comparison to the vehicle-treated group (p<0.05 and p<0.01; Fig. 7E). Similarly, the protein expression of Bcl-2 was markedly decreased (p<0.01; Fig. 9E), Bax protein expression was significantly increased (p<0.01; Fig. 8E), and the Bcl-2/Bax ratio was significantly decreased (p<0.01; Fig. 9F) in vehicle-treated group compared to the sham-operated group. However, the Bcl-2/Bax ratio returned to approximately normal levels (those of the control) in the groups pre-treated with Mat (H) and nimodipine (p<0.01; Fig. 9F).

## Discussion

In the present study, we provide evidence that Mat is an effective neuroprotectant against focal cerebral ischemia. We demonstrated that pre-treatment with Mat reduced the infarct volume, improved neurological deficits and alleviated histopathology and morphological injury in mice subjected to MCAO, which indicated that Mat has the ability to protect the mouse brain against I/R injury. Moreover, we explored the mechanisms responsible for the neuroprotective effects of Mat against focal cerebral ischemia by determining the levels of oxidative stress and apoptotic biomarkers in the ischemic brain. Our data demonstrated that the neuroprotective effects of Mat may be associated with the suppression of the cell apoptosis through the regulation of the expression of Bax, Bcl-2 and caspase-3 proteins and an increase in the Bcl-2/Bax ratio, as well as the suppression of oxidative stress, as evidenced by a decrease in the MDA content, and an increase in SOD, GSH-Px and CAT activity and T-AOC in mice subjected to MCAO.

MCAO is a classical stroke model of temporary regional ischemia and is considered reliable and less invasive (21,23,24); it is extensively used to study neurological, histopathological and biochemical changes and the mechanisms of cerebral ischemic injury in mice. The infarct volume and neurological deficit score play important roles in evaluating the validity of cerebrovascular drugs in the treatment of ischemic brain disease. In the present study, pre-treatment with Mat significantly decreased the percentage of the brain infarct volume and improved neurological deficit scores at 24 h following ischemia in a mouse model of cerebral infarction. In addition, the degree of ischemic damage was observed by H&E staining, a method commonly used to identify the histopathological changes associated with the development of I/R injury (25). Our results demonstrated that the size of the ischemia-affected regions and neuronal necrosis were significantly decreased by pre-treatment with Mat. Another important observation was shown by electron microscopy; the cortex neurons showed prominent morphological injuries in the mice following MCAO. However, these morphological changes and damage were mitigated in the group pre-treated with Mat. Therefore, the results from behavioral and morphological analysis suggested that pre-treatment with Mat exerted a protective effect against cerebral I/R injury.

Ischemic stroke remains an urgent public health concern and is the major cause of mortality and permanent neurological disability worldwide (26). During the past two decades, accumulating research into the complex mechanisms of ischemic stoke has indicated that excessive reactive oxygen species (ROS) production and subsequent oxidative stress play harmful roles during cerebral I/R injury (27,28). Along with the occurrence and development of reperfusion, multifarious pernicious processes, including the overproduction of oxygen radicals, the inactivation of detoxification systems, the consumption of antioxidants and the failure to adequately replenish antioxidants occur in ischemic brain tissue (29), which contributes to oxidative damage to cellular macromolecules such as lipids, proteins and nucleic acids in the ischemic tissue, leading to membrane damage, cell death and brain dysfunction (30,31). As the toxic final product of lipid peroxidation, MDA is a sensitive marker of oxidative stress and is responsible for cytotoxic effects and neuronal death (32). An efficient antioxidant defense system involving endogenous antioxidant enzymes, such as SOD, GSH-Px and CAT (33) plays an important role in the maintenance of low concentrations of oxidants and redox homeostasis in tissue through the scavenging of oxidants, preventing deleterious ROS generation (34). In the present study, we demonstrated that pre-treatment with Mat for 7 consecutive days significantly increased SOD, GSH-Px, CAT and activity, and T-AOC, and decreased MDA levels in a dose-dependent manner. These results suggest that Mat protects the brain from cerebral I/R injury by exerting antioxidant effects.

On the other hand, apoptosis, a form of programmed cell death characterized by DNA fragmentation (35), is now being regarded as a key event in the acceleration of tissue injury and cell death following cerebral ischemia (36). As an actively regulated form of cell death, apoptosis is mediated by two pathways following cerebral ischemia: the intrinsic and extrinsic pathways (37). The intrinsic pathway originates from the mitochondrial release of cytochrome *c*. The release of cytochrome *c* into the cytosol promotes the formation of the apoptosome, a complex composed of the apoptotic protease activating factor-1 (Apaf-1), procaspase-9 and ATP (38). The apoptosome permits the autoactivation of procaspase-9, which is followed by the activation of procaspase-3. Ultimately, the activation of caspase-3 leads to DNA fragmentation (39). In present study, the results of H&E staining and transmission electron microscopy indicated that apoptotic morphological characteristics, such as the breakup of the nuclear membrane, pyknosis of the nucleolus and the disruption of the mitochondrial ridge were evident in the neurons following cerebral ischemia. Pre-treatment with Mat alleviated these effects.

It is well known that caspases are a family of intracellular cysteine proteases involved in the initiation and execution of cell apoptosis (40). Sudies have identified that caspase-3 is a potent, terminal caspase that plays a crucial role in executing apoptosis through the mitochondrial-dependent pathway (41) and increased neuronal caspase-3 expression has been observed in transient ischemic injury (42). Moreover, the Bcl-2 family proteins, composed of pro-apoptotic and anti-apoptotic members, are vital to the intrinsic apoptotic pathway and control the activation of downstream caspases (43). Further evidence of the crucial role of Bcl-2 family proteins in neuronal cell death has been provided by recent studies on cerebral ischemia in rats, showing that the dysregulation of the Bcl-2 family proteins exacerbates ischemic neuronal injury (5,44,45). As a member of the Bcl-2 family, the anti-apoptotic protein, Bcl-2, is localized to the mitochondrial membrane, helping maintain membrane integrity and preventing cytochrome *c* from being released into the cytoplasm, which is a central step in the apoptotic process (36). By contrast, the pro-apoptotic protein, Bax, is a cytoplasmic protein, and when cells are exposed to various apoptotic stimuli, the protein translocates specifically to the mitochondria, which causes the disequilibrium between Bax and Bcl-2 (46). This disequilibrium leads to mitochondrial permeability changes and promotes the release of cytochrome *c* from the mitochondria to cytoplasm. The subsequent activation of caspase-3 eventually leads to cerebral ischemia-induced apoptosis (47). In the present study, we demonstrated that the decrease in the expression of Bax and caspase-3 and the concurrent increase in the expression of Bcl-2 and the Bcl-2/Bax ratio by 7 days of pre-treatment with Mat (30 mg/kg), which strongly favors the notion that Mat has anti-apoptotic activity as recently reported (22), occurred least in part, through the modulation of the Bcl-2/Bax ratio and the inhibition of caspase-3 expression. Increasing evidence indicates that Mat induces apoptosis in a number of cancer cell lines (12,48–50). The dual regulation and control of Mat as regards apoptosis may be due to its differential effects on dividing cells and non-dividing cells.

In this study, focal cerebral ischemia reperfusion was evident by 2 h of MCAO and reperfusion for 24 h in mice. However, the therapeutic window of Mat is and its maximal protective effects against cerebral I/R injury were not sufficiently elucidated. A major limitation of the present study was that multiple pathways are involved in the apoptotic process following cerebral I/R injury and additional investigations are required to determine the detailed molecular targets mediating the neuroprotective effects of Mat.

In conclusion, this study demonstrated that Mat exerted neuroprotective effects against cerebral I/R injury mice. Mat significantly improved neurological deficits, reduced the infarct volume and the percentage of apoptotic neurons, and inhibited histopathologicla and morphological changes. Taken together, the findings of this study suggest that the mechanisms underlying the neuroprotective effects of Mat are associated with its antioxidant and anti-apoptotic properties by targeting the apoptosis-related proteins, caspase-3, Bax and Bcl-2. Therefore, Mat may be used as an effective neuroprotective agent for the treatment of stroke in clinical trials.
